# Upper arm versus forearm transcutaneous oximetry during upper limb abduction in patients with suspected thoracic outlet syndrome

**DOI:** 10.3389/fphys.2022.1033137

**Published:** 2022-11-08

**Authors:** Jeanne Hersant, Simon Lecoq, Pierre Ramondou, Mathieu Feuilloy, Pierre Abraham, Samir Henni

**Affiliations:** ^1^ Vascular Medicine, University Hospital, Angers, France; ^2^ UMR CNRS 1083, INSERM 6015, LUNAM University, Angers, France; ^3^ School of Electronics (ESEO), Angers, France; ^4^ UMR CNRS 6613, LAUM, Le Mans, France; ^5^ Sports and Exercise Medicine, University Hospital, Angers, France

**Keywords:** thoracic outlet syndrome, transcutaneous oximetry, pathophysiology, artery, ischemia, upper limb, peripheral artery disease, diagnosis

## Abstract

**Context:** Thoracic outlet syndrome (TOS) is common among athletes and should be considered as being of arterial origin only if patients have “clinical symptoms due to documented symptomatic ischemia.” We previously reported that upper limb ischemia can be documented with DROPm (minimal value of limb changes minus chest changes) from transcutaneous oximetry (TcpO2) in TOS.

**Purpose:** We aimed to test the hypothesised that forearm (F-) DROPm would better detect symptoms associated with arterial compression during abduction than upper arm (U-) DROPm, and that the thresholds would differ.

**Methods:** We studied 175 patients (retrospective analysis of a cross-sectional acquired database) with simultaneous F-TcpO2 and U-TcpO2 recordings on both upper limbs, and considered tests to be positive (CS+) when upper limb symptoms were associated with ipsilateral arterial compression on either ultrasound or angiography. We determined the threshold and diagnostic performance with a receiver operating characteristic (ROC) curve analysis and calculation of the area under the ROC curve (AUROC) for absolute resting TcpO2 and DROPm values to detect CS+. For all tests, a two-tailed *p* < 0.05 was considered indicative of statistical significance.

**Results:** In the 350 upper-limbs, while resting U-TcpO2 and resting F-TcpO2 were not predictive of CS + results, the AUROCs were 0.68 ± 0.03 vs. 0.69 ± 0.03 (both *p* < 0.01), with the thresholds being −7.5 vs. −14.5 mmHg for the detection of CS + results for U-DROPm vs. F-DROPm respectively.

**Conclusion:** In patients with suspected TOS, TcpO2 can be used for detecting upper limb arterial compression and/or symptoms during arm abduction, provided that different thresholds are used for U-DROPm and F-DROPm.

**Clinical Trial Registration:** ClinicalTrials.gov, identifier NCT04376177.

## Introduction

Thoracic outlet syndrome (TOS) is common among those engaged in professional activities involving repetitive arm movements as well as among athletes (Chandra et al., 2014; Shutze et al., 2017; Hilberg et al., 2021). TOS is reported to occur in almost half of vascular diseases in sports (Arko et al., 2001). The diagnosis of thoracic outlet syndrome (TOS) remains difficult (Demondion et al., 2006; Ammi et al., 2017; Ahmad and Murthy, 2018; Burt, 2018; Illig, 2018). Surgical treatment of TOS is a significant source of malpractice claims against vascular surgeons (Li and Dissanaike, 2021). Consequently, documenting ischemia in cases of compression (impaired inflow) of the subclavian artery is of major interest in patients with TOS, even if symptoms are generally considered to be of neural origin.

According to the standards proposed for thoracic outlet syndrome by the Society for Vascular Surgery, TOS should be considered as being of arterial origin only if patients have “clinical symptoms due to documented symptomatic ischemia or objective subclavian artery damage caused by compression” (Illig et al., 2016). We assume that transcutaneous oxygen pressure (TcpO2) could fill a gap and provide objective arguments for the presence of ischemia in patients with suspected TOS.

Among positional manoeuvres aiming to induce a compression of the neurovascular bundle, the Roos test (also referred to as the elevated arm stress test: EAST) is the most widely used provocative manoeuvre. The test consists of abduction and external rotation, also known as the surrender/candlestick position (Nord et al., 2008; Fried and Nazarian, 2013; Adam et al., 2018). We recently showed that during the Roos test in TOS, the forearm ischemia resulting from impaired positional perfusion to the upper limb can be estimated by TcpO2 bilaterally and simultaneously (Henni et al., 2019; Abraham et al., 2020). TcpO2 has been used for years at the lower limb level in patients with arterial disease (Abraham et al., 2018a; Constans et al., 2019). In patients with suspected critical lower limb ischemia, when TcpO2 remains within normal limits at rest, elevation of the limb allows for better discrimination between normal and abnormal TcpO2 results. Indeed, when perfusion is impaired but TcpO2 at rest remains within normal values, the post-stenotic perfusion pressure is insufficient to compensate for the hydrostatic pressure decrease that is induced by limb elevation. As a result, TcpO2 decreases more in impaired perfused limbs than in normally perfused limbs where only a moderate decrease is observed as a result of decreased hydrostatic pressure (Paraskevas et al., 2006; Ruangsetakit et al., 2010). Maximal amplitude of the decrease in TcpO2 can be expressed as the “minimal decrease from rest of oxygen pressure” (DROPm) index (Henni et al., 2019; Abraham et al., 2020). We also previously showed that positional compression of the subclavian artery does not necessarily result in arterial ischemia (Hersant et al., 2022).

We then hypothesised that similarly to what is observed in the lower limb, elevation of the upper limb would facilitate the detection of impaired but non-abolished perfusion. If so, the position of TcpO2 probes on the upper limb could be important in the evaluation of ischemia because there is a wide difference in the amplitude of elevation at the level of the forearm (F-), as compared to the upper arm (U-) in the candlestick/surrender position. We hypothesised that the U-DROPm value would be higher than the F-DROPm value during manoeuvres potentially inducing arterial compression. We also hypothesised that F-DROPm would better predict the presence of positional arterial compression of the subclavian artery and/or positional upper limb symptoms than U-DROPm, but with different cut-off values for F-DROPm compared to U-DROPm. To test these hypotheses, we performed a cross-sectional study of patients referred for suspected TOS by simultaneously recording U-TcpO2 and F-TcpO2 during a standardised provocative test. Because no ideal gold standard exists for diagnosing TOS, we compared TcpO2 results to symptoms only, ultrasound only, angiography only and combinations of these three elements.

## Materials and methods

### Experimental design

A cross-sectionnal study was performed among all patients who were referred to our laboratory for symptoms suspected as resulting from TOS between 1 January 2019, and 31 May 2021. TcpO2 has become routine in our medical practice as a way to demonstrate the presence of regional blood flow impairment during provocative manoeuvres (Abraham et al., 2020). All patients were recorded in a clinical database that received full administrative agreement. Patients who objected to the use of their data, who were unable to understand the information due to linguistic or cognitive reasons, or who were under 18 years of age were not included in the database. As an observation of our medical routine and according to French law, no individual consent was required but all the patients were informed that they could object to the use of their medical file for research purposes. Our Institutional Ethics Committee approved the retrospective and prospective analysis of the database of the “Clinical Routine in Thoracic Outlet Syndrome” (SKIPA) study under reference 2020/17 and registered on ClinicalTrials.gov under reference NCT04376177. The authors certify that the study was performed in accordance with the ethical standards as laid down in the 1964 Declaration of Helsinki and its later amendments or comparable ethical standards. As routine practice during each visit, we recorded the patient’s demographics and conditions, including age, sex, weight, height, history of chest, shoulder or arm trauma or surgery, and any ongoing treatments. Patients self-completed the “disability of the arm and shoulder”. The DASH includes 30 items (plus eight optional items at the end of the questionnaire) and can be calculated only if 27 of the first 30 items are available (presence of an answer and no duplicate answer to the same item), For the DASH, a score of 0 is no disability and 100 is complete inability (Hudak et al., 1996). Note that approximately half of the 76 patients in our recent publication (Abraham et al., 2020) had simultaneous arm and forearm recordings and are included in the 175 patients reported in the present manuscript, with permission from IOS Press.

### Ultrasound and radiological imaging

Ultrasound investigations and/or angiographies were performed by trained operators who were independent from the operators who performed the TcpO2 recordings and who were blinded to the TcpO2 results. The physicians were free to perform any kind of manoeuvre that they required to try to induce an arterial compression, but all tests included at least the candlestick procedure. Results from the ultrasound investigations and angiography were retrieved from the physician’s report, when performed within 6 months and 1 year respectively from TcpO2 recordings and encoded, arm by arm, as either positive (+) or negative (−) for the presence of arterial compression by ultrasound (US), angiography (Ang) or imaging (Imag). For imaging, the presence of a compression (Imag+) during provocative manoeuvres of the subclavian artery was defined as ultrasound (US+) OR angiography (Ang+), while Imag- was both US- and Ang-.

### TcpO2 recordings

**FIGURE 1:**
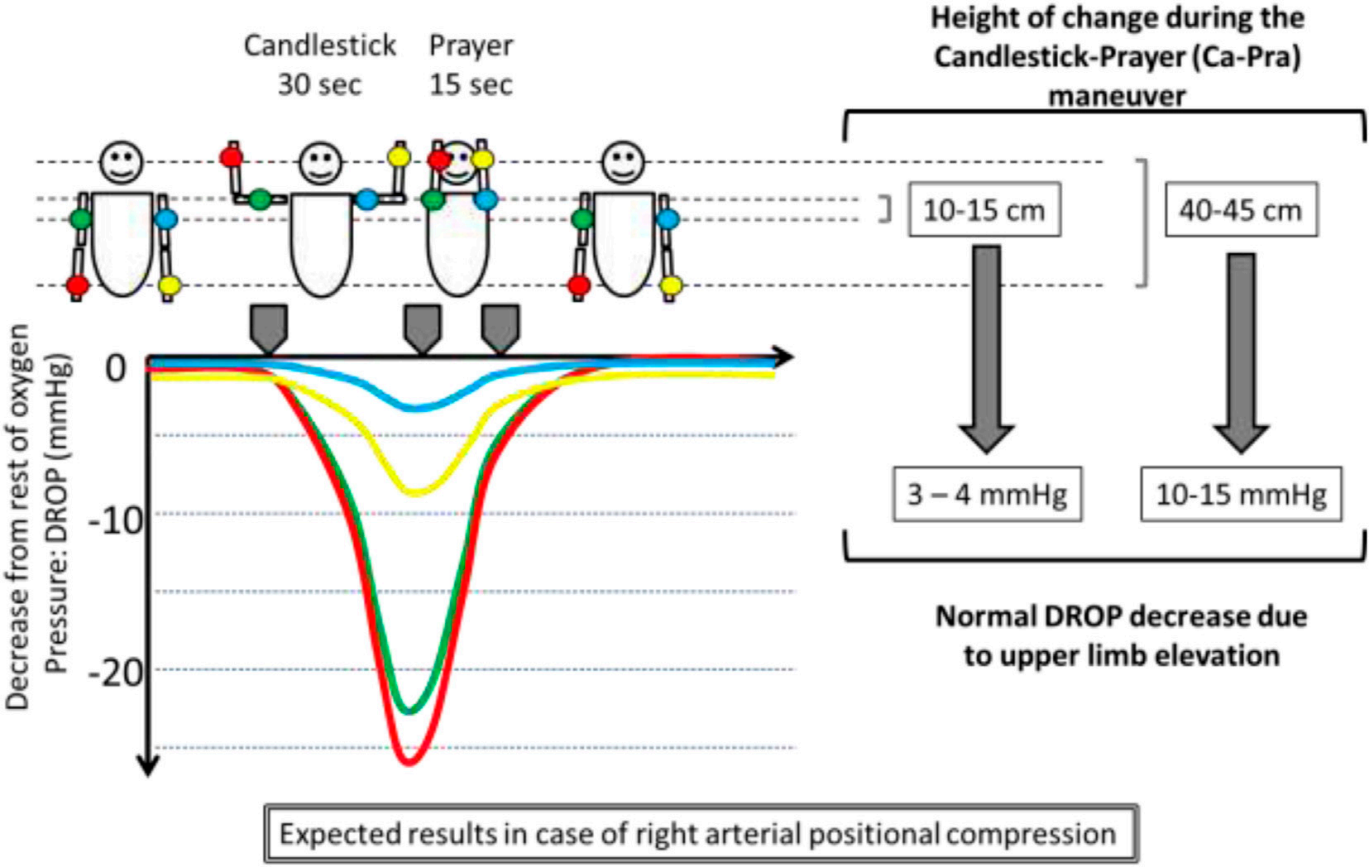
Illustration of a unilateral right arterial compression and of the effect of changes in the position of the probes relative to heart level (on the left side of the subject) on TcpO2 values expressed as decrease from rest of oxygen pressure (DROP: limb changes from rest minus chest changes from rest). DROPm is the minimal value observed on the DROP curve.

TcpO2 recordings were performed using a TCM400 (Radiometer, DK) with E5250 probes, blinded to the results of ultrasound imaging. The TCM400 device can receive a signal from up to six probes. Double calibration against air was performed before each recording session. Once the system was calibrated, when five probes were available, probes were positioned on the dorsal aspect of each distal third of the forearm (F-), and each mid third of the upper arm (U-) with the patient standing still. The fifth probe was used as a reference on the chest. After probe positioning, a 15- to 20-min heating period was observed to heat the skin to 44°C and reach stable calibration values. The recording of TcpO2 absolute values was carried out using a software program that automatically calculates the decrease from rest of oxygen pressure (DROP) values. TcpO2 changes are calculated at the chest and limb levels, with the resting value considered zero. DROP corresponds to the subtraction of the chest TcpO2 changes from each of the upper limb TcpO2 changes. The resulting DROP is expressed in millimetres of mercury (mmHg) (Abraham et al., 2018b). DROP calculation allows for eventual systemic pO2 changes to be taken into account and allows for elimination of error due to the unpredictable transcutaneous gradient. DROP calculation was shown to be reliable for intra-test and test-retest recordings (Henni et al., 2018). In the absence of ischemia and gravity changes, DROPm should remain close to zero throughout provocative manoeuvres. Furthermore, in the absence of limb ischemia, DROP shall moderately decrease in case of changes in hydrostatic pressure (DROPm remaining close to zero), whereas ischemia should result in an ample decrease of DROP (lower DROPm values) with a progressive return to zero value after ischemia is released. A schematic representation of changes expected from hydrostatic pressure and from the presence of an impaired arterial inflow is presented in Figure 1.

In brief, DROPm being a negative value, a lower DROPm value represents a more severe haemodynamic or ischemic response.

### Provocative manoeuvres

All tests started with a 1-min recording at rest and TcpO2 was recorded throughout and for at least 1 min after the end of the provocative manoeuvre. For the analysis, the minimal DROP (DROPm) at the upper arm (U-DROPm) and forearm (F-DROPm) level during or following each manoeuvre was retained for the analysis. Note that as we share probes between two different TCM400 systems in our laboratory, not all patients could have recordings taken with five probes and were then excluded from the study. The provocative test in our routine was a Candlestick-Prayer (Ca-Pra) manoeuvre (Hersant et al., 2021a; Hersant et al., 2021b). The Ca-Pra manoeuvre is a modified version of the Roos test (or elevated arm stress test: EAST) during which the candlestick/surrender position (Ca) lasts only 30 s, after which the patients move their elbow forward without lowering their hands as if praying (Pra) before the arms are lowered. The specific interest of the Ca-Pra manoeuvre in the present study is to standardise the duration of abduction. For each provocative manoeuvre, we recorded the presence (Symp+) or absence (Symp−) of positional symptoms in each arm. Transient pain during elevation disappearing in the immobile abduction position were not considered as an indication of potential neurovascular compression. Also note that contrary to the Roos/EAST test, patients were not asked to open and close their hands during abduction.

### Statistical analysis

We encoded the presence of a compression on imaging associated with symptoms during the manoeuvre as CS+, whereas absence of symptoms and compression, symptoms without compression, or compression without symptoms, was encoded as CS−. Distribution of TcpO2 and DROPm values was tested using the Kolmogorov-Smirnov test and the data are presented as numbers (percentages), medians [25°/75° centiles], or means ± standard deviations (SD) according to normal or non-normal distribution. Between-arm differences were calculated using Chi-squared and paired t-tests (or the Wilcoxon test). A receiver operating characteristic (ROC) curve analysis was performed to determine the diagnostic performance of each parameter. Determination of the area under the ROC curve (AUROC) was used to estimate the diagnostic performance of F-TcpO2, U-TcpO2, F-DROPm, and U-DROPm for each end point used (symptoms, ultrasound, imaging, etc … ). It is generally considered that the test is good or excellent for AUROC values above, fair or poor for AUROC values between 0.7–0.8 and between 0.6–0.7 respectively, and failed for AUROC values between 0.5–0.6 (El Khouli et al., 2009). A comparison of AUROC between F-DROPm and U-DROPm and between F-TcpO2, U-TcpO2 was performed with the method described by Hanley and McNeil (Hanley and McNeil, 1982). The optimal cut-off point to be used at the arm and forearm level to argue for Symp+, US+, Ang+, Imag+, and CS + results was calculated as the lowest distance of the ROC curve to the 100% sensitivity/100% specificity angle. This lowest distance is considered to define the threshold value that provides the best compromise of sensitivity and specificity for an equal cost of false positive and false negative results. Using each determined value we calculated the sensitivity, specificity, positive and negative predictive values, positive and negative likelihood ratios, and accuracy. All statistical analyses were performed using SPSS (IBM SPSS statistics V15.0, Chicago, IL, United States). For all tests, a two-tailed *p* < 0.05 was considered indicative of statistical significance.

## Results

**FIGURE 2:**
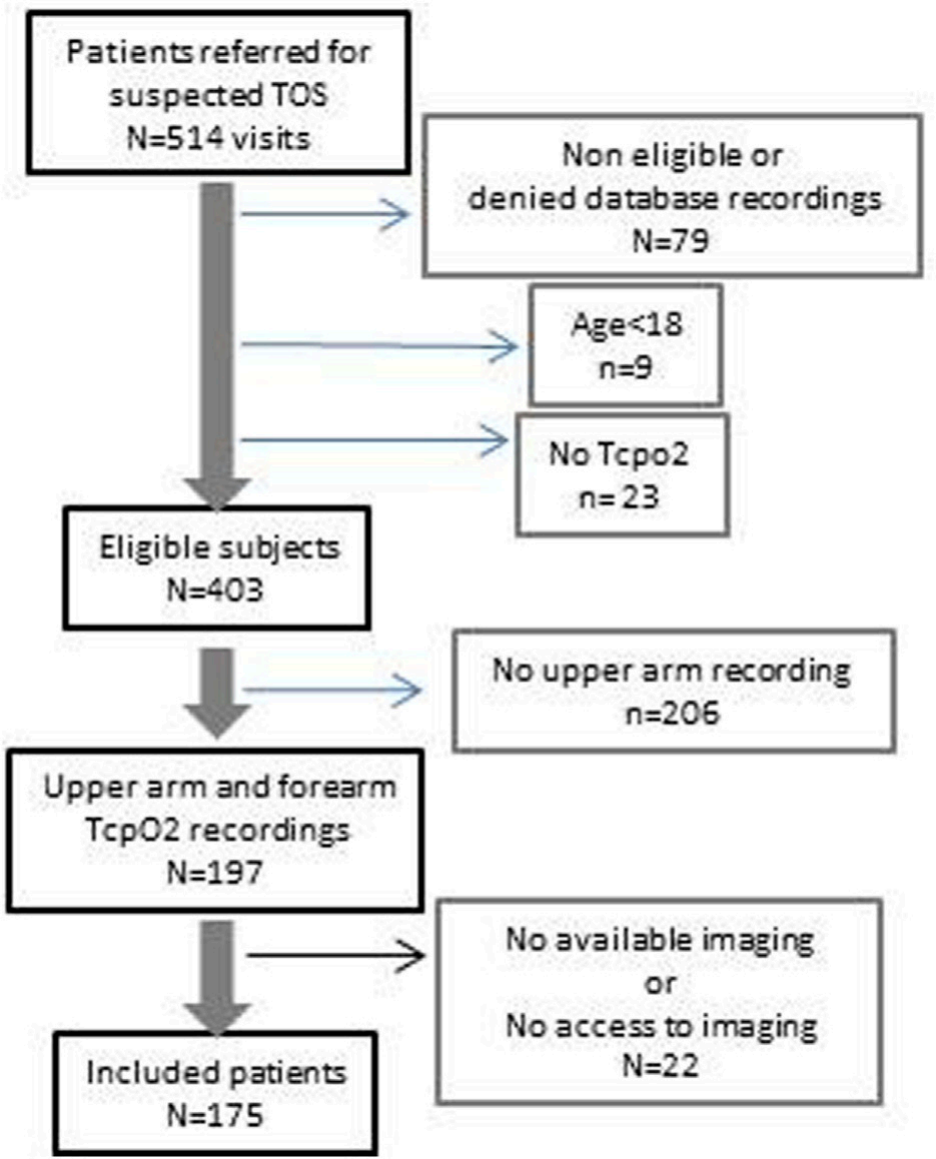
Flowchart of the study.

Among the 514 referred patients, only 175 subjects (350 arms) were eligible, had accessible or available imaging results, and had simultaneous arm and forearm TcpO2 recordings as shown in Figure 2. One hundred and three of these patients engaged in repetitive movements in the course of their professional activities or in weight training. Only 62 subjects were involved in regular sports activities including running (*n* = 32), cycling (*n* = 7), weightlifting (*n* = 4), and badminton (*n* = 3), and most had to stop doing sports because of their upper limb pain.

Among the studied subjects, 58 were male (33.1%). The subjects were 40.7 ± 12.1 years old, with a weight of 72.8 ± 15.9 kg and height of 167 ± 9 cm. Most subjects were right-handed (*n* = 153, 87.4%) and had displayed symptoms for a minimum of 6 months (*n* = 159, 90.9%). 94 patients took painkillers for their upper limb pain (*n* = 94, 53.7%). The disability of arm and shoulder (DASH) score was available (at least 27 answers) for 128 patients and was 41 ± 21. According to their history, patients complained of right (*n* = 57), left (*n* = 48) or bilateral (*n* = 70) positional symptoms. During the Ca-Pra manoeuvre, patients complained of right (n = 41), left (*n* = 46) or bilateral (*n* = 61) symptoms, but 37 patients remained asymptomatic.

All but two patients had ultrasound investigations and only 37 patients had an angiogram as a pre-surgical investigation. Results for arterial pressure, ultrasound, angiography and transcutaneous oximetry at rest or during the provocative manoeuvre are shown in Table 1.

**TABLE 1:** Results of investigations among the studied patients. Positive imaging is either positive ultrasound or positive angiography. *p* is the difference between right and left side regardless of the presence or absence of symptoms.

Recorded parameter	Right arm		Left arm		*p*
	No symptoms during the Ca-Pra test	Symptoms during the Ca-Pra test	No symptoms during the Ca-Pra test	Symptoms during the Ca-Pra test	
Systolic arterial pressure (mmHg)	135 ± 15	133 ± 14	135 ± 14	132 ± 14	0.548
Diastolic arterial pressure (mmHg)	82 ± 11	82 ± 12	84 ± 12	81 ± 10	0.585
Positive/available ultrasound	18/73 (24.7%)	39/100 (39.0%)	26/78 (33.3%)	42/95 (44.2%)	0.218
Positive/available angiography	7/13 (53.8%)	14/24 (58.3%)	10/15 (66.7%)	18/22 (81.8%)	0.085
Positive/available imaging	23/73 (31.5%)	45/102 (44.1%)	29/78 (37.2%)	49/97 (50.5%)	0.278
Chest TcpO2 (mmHg)	68.5 ± 11.9				
Upper arm TcpO2 (mmHg)	70.3 ± 11	71.3 ± 10	70.6 ± 12	68.3 ± 12	0.061
Forearm TcpO2 (mmHg)	70.9 ± 11	72.9 ± 11	70.7 ± 10	69.6 ± 11	0.008
Upper arm DROPm (mmHg)	−3 [−8/−1]	−6 [−14/−2]	−4 [−8/−1]	−6 [−18/−2]	0.428
Forearm DROPm (mmHg)	−12 [−16/−7]	−16 [−22/−8]	−10 [−15/−6]	−13 [−23/−8]	0.245

**FIGURE 3:**
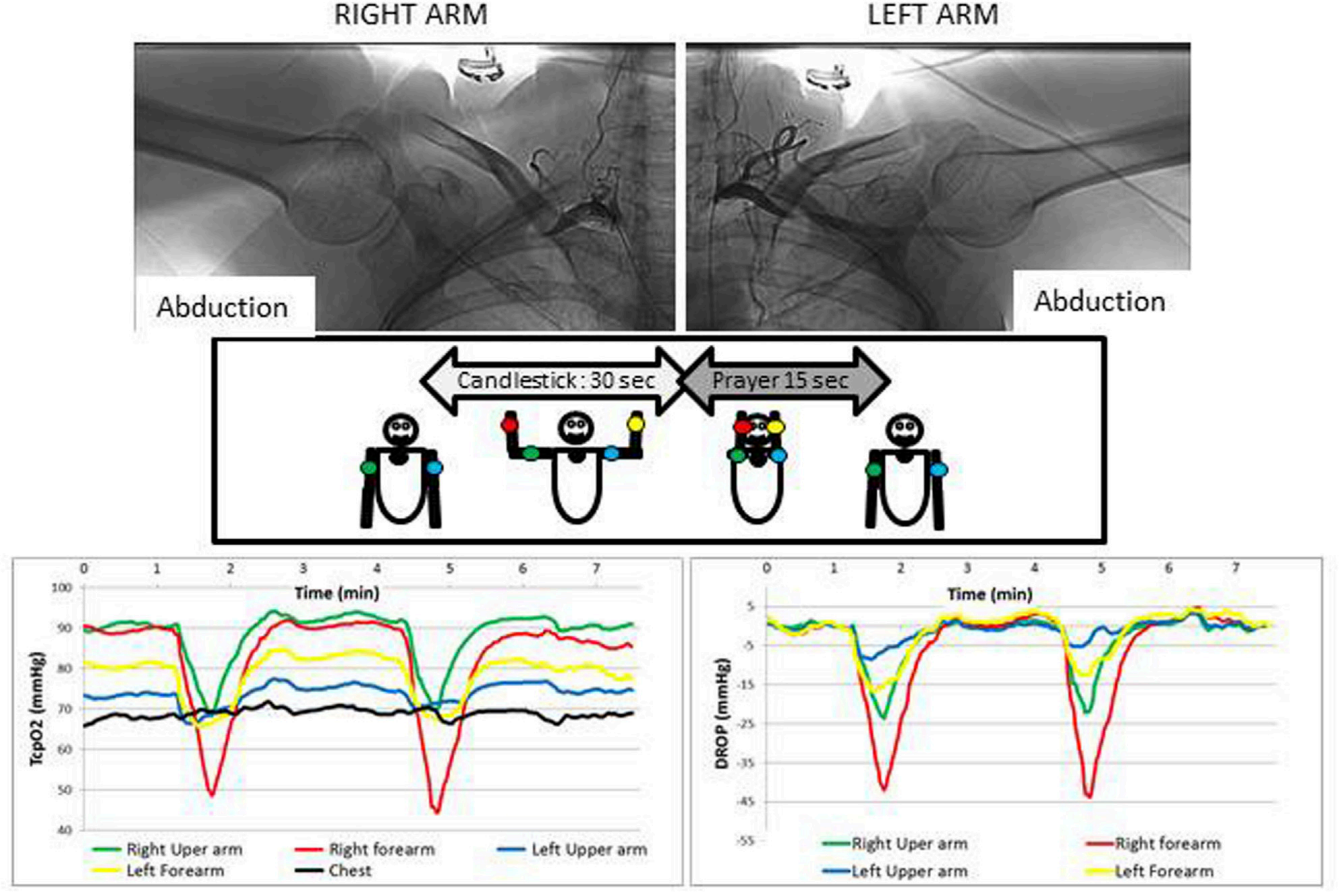
Typical example of a patient complaining of bilateral paraesthesia and pain during arm elevation. As shown, an arterial compression is observed during arteriography (upper panels), but whereas it is occlusive on the right side, on the left side the compression is not occlusive and a significant collateral circulation exists that likely explains why the decrease in DROP values is much more important on the right side than on the left. It is interesting to note that absolute values for TcpO2 (left panel) are very different at the different probe positions and that DROP values (lower right panel) start to increase from the beginning of the prayer position, confirming that the decrease does not result solely from arm elevation. Also note that in this case the Ca-Pra procedure was performed twice, confirming the reliability of the results observed.

A typical example of a recording and the corresponding angiography in a patient showing unilateral positional occlusion of the subclavian artery is reported in Figure 3.

**FIGURE 4:**
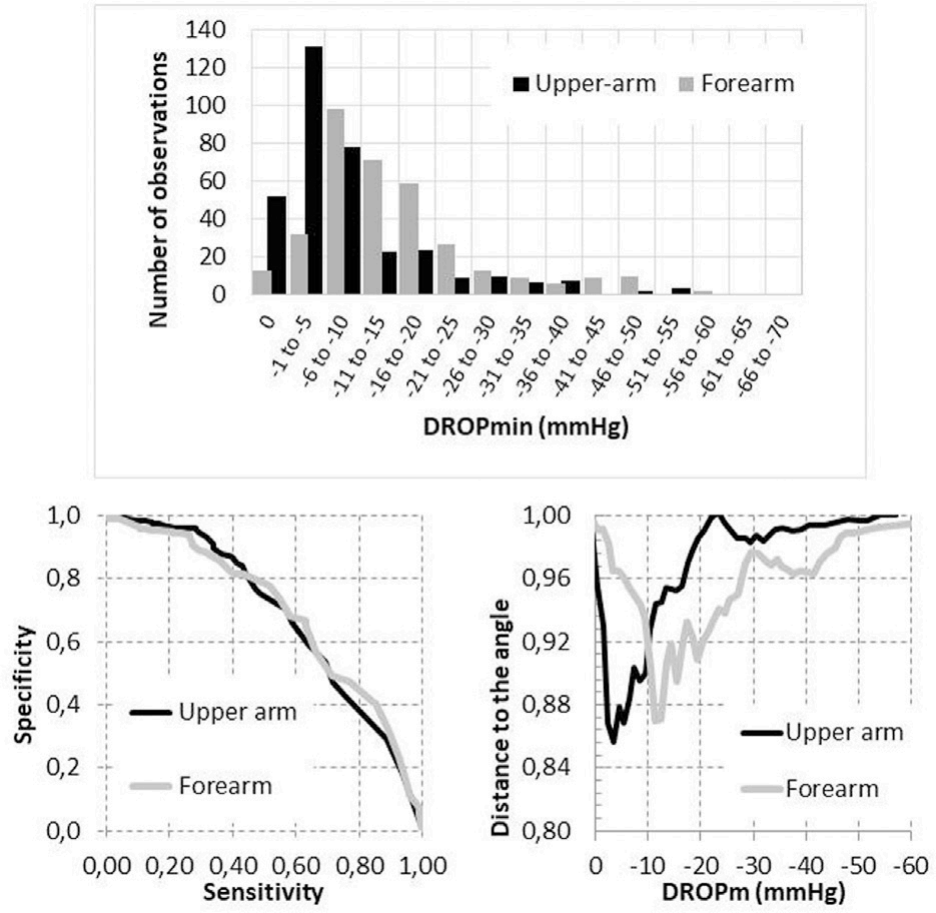
Distribution of DROPm values observed at the forearm and upper arm level (upper panel) and receiver operating characteristics of the DROPm values to detect the presence of compression on imaging (either ultrasound or angiography) with associated positional symptoms (lower left panel) and distance to the 100% sensitivity/100% specificity angle as a function of the DROPm value at the upper arm and forearm levels (lower right panel).

The Kolmogorov-Smirnov test confirmed the normal distribution of resting U-TcpO2 (*z* = 1.354; *p* = 0.051) and F-TcpO2 (*Z* = 0.865; *p* = 0.442) values. No significant difference was found in relation to absolute value for resting U-Tcpo2 (70.1 ± 11.2 mmHg) and F-TcpO2 (71.1 ± 10.8 mmHg (*p* = 0.111). On the contrary, as shown in Figure 4, there was a non-gaussian distribution of U-DROPm (*Z* = 4.065; *p* < 0.001) and F-DROPm (*z* = 2.973; *p* < 0.001) values, with a median value of −5 [−11/2] mmHg and −12 [−19/−7] mmHg among the 350 upper arm and 350 forearm values respectively (*p* < 0.001).

On a limb-by-limb basis, as reported in Tables 2, 3, the absolute TcpO2 value at rest was not predictive of the presence of Symp+, US+, Ang+, Imag + or CS+, whereas all areas (except F-DROPm for Ang+) were significant for DROPm results with areas ranging from 0.596 to 0.689.

**TABLE 2:** Values for transcutaneous oximetry at rest (TcpO2) and during the Ca-Pra maneuvre (DROPm) at the forearm and upper arm levels, for detecting the presence of symptoms (Symp+), a compression during ultrasound (US+), during angiography (Ang+), or during imaging (Img+): either a positive ultrasound or a positive angiography, or symptoms with positive imaging (CS+). Note that for ultrasounds and angiography, the numbers of observations are only 346 and 74 respectively. Numbers in parentheses are the number of upper limbs in each group. *p* for + vs. – is comparison of values in patients with positive or negative results to the reference technique.

Results for symptoms (*n* = 350)	Value in Symp+(*n* = 199)	Value in symp− (*n* = 151)	*p* For + vs. −
U-TcpO2	69.8 ± 10.9	70.5 ± 11.5	0.58
F-TcpO2	71.3 ± 10.9	70.8 ± 10.7	0.67
U-DROPm	−6 [−16; −2]	−4 [−8; −1]	0.01
F-DROPm	−15 [−23; −8]	−11 [−16; −6]	0.01
**Results for ultrasound (*n* = 346)**	**Value in US+(*n* = 125)**	**Value in US-(*n* = 221)**	***p* for + vs. −**
U-TcpO2	69.6 ± 11.2	70.6 ± 11.1	0.48
F-TcpO2	70.6 ± 10.9	71.5 ± 10.7	0.48
U-DROPm	−7 [−20; −2]	−4 [−8; −1]	0.01
F-DROPm	−16 [−25; −10]	−11 [−16; −7]	0.01
**Results for angiography (*n* = 74)**	**Value in Ang+(*n* = 49)**	**Value in Ang-(*n* = 25)**	***p* for + vs. −**
U-TcpO2	69.1 ± 13.0	70.1 ± 12.5	0.75
F-TcpO2	70.2 ± 11.4	72.5 ± 10.6	0.41
U-DROPm	−9 [−19; −2]	−6 [−7; −2]	0.04
F-DROPm	−15 [−22; −10]	−13 [−18; −7]	0.18
**Results for imaging (*n* = 350)**	**Value in Imag+(*n* = 146)**	**Value in Imag-(*n* = 204)**	***p* for + vs. −**
U-TcpO2	69.1 ± 11.6	70.9 ± 10.9	0.14
F-TcpO2	70.6 ± 10.6	41.4 ± 10.9	0.50
U-DROPm	−8 [−18; −2]	−4 [−8; −1]	0.01
F-DROPm	−16 [−24; −10]	−11 [−16; −7]	0.01
**Results for imaging and symptoms (*n* = 350)**	**Value in CS+(*n* = 95)**	**Value in CS-(*n* = 255)**	***p* for + vs. −**
U-TcpO2	68.8 ± 11.2	70.6 ± 11.2	0.20
F-TcpO2	71.6 ± 11.2	70.9 ± 10.7	0.60
U-DROPm	−8 [−25; −3]	−4 [−8; −1]	0.01
F-DROPm	−18 [−30; −11]	−12 [−17; −7]	0.01

**TABLE 3:** Area under the ROC curve for transcutaneous oximetry at rest (TcpO2) and during the Ca- Pra maneuvre (DROPm) at the forearm and upper arm levels, for detecting the presence of symptoms (Symp+), a compression during ultrasound (US+), during angiography (Ang+), or during imaging (Img+): either a positive ultrasound or a positive angiography, or symptoms with positive imaging (CS+). Note that for ultrasounds and angiography, the numbers of observations are only 346 and 74 respectively. Numbers in parentheses are the number of upper limbs in each group. *p* for the area is comparison from a random choice.” *p* for U vs. F is the comparison between upper-arm and forearm results.Sensit. Specif. PPV, NPV, LR+, LR− and Accur., are for sensitivity (in %), specificity (in %), positive predictive value (in %), negative predictive value (in %), positive likelihood ratio, negative likelihood ration and accuracy (in %), respectively.

Results for symptoms (*n* = 350)	Area ± SD of ROC curve	*p* For the area	*p* For U vs. F	Sensit.	Specif.	PPV	NPV	LR+	LR−	Accur.
U-TcpO2	0.48 ± 0.03	0.50	0.29	50.8	53.6	59.1	45.3	1.09	0.92	52.0
F-TcpO2	0.53 ± 0.03	0.43		52.3	45.7	55.9	42.1	0.96	1.04	49.4
U-DROPm	0.62 ± 0.03	<0.01	0.64	59.8	57.0	64.7	51.8	1.39	0.71	58.6
F-DROPm	0.64 ± 0.03	<0.01		51.3	71.5	70.3	52.7	1.80	0.68	60.0
**Results for ultrasound (*n* = 346)**	**Area ± SD of ROC curve**	***p* For the area**	***p* For U vs. F**	**Sensit.**	**Specif.**	**PPV**	**NPV**	**LR+**	**LR−**	**Accur.**
U-TcpO2	0.48 ± 0.03	0.54	0.98	40.0	62.4	37.6	64.8	1.07	0.96	54.3
F-TcpO2	0.48 ± 0.03	0.56		37.2	65.0	54.4	48.0	1.06	0.97	50.3
U-DROPm	0.65 ± 0.03	<0.01	0.86	64.8	55.2	45.0	73.5	1.45	0.64	58.7
F-DROPm	0.66 ± 0.03	<0.01		58.4	69.2	51.8	74.6	1.90	0.60	65.3
**Results for angiography (*n* = 74)**	**Area ± SD of ROC curve**	***p* for the area**	***p* for U vs. F**	**Sensit.**	**Specif.**	**PPV**	**NPV**	**LR+**	**LR−**	**Accur.**
U-TcpO2	0.48 ± 0.07	0.81	0.81	55.1	44.0	65.9	33.3	0.98	1.02	51.4
F-TcpO2	0.46 ± 0.07	0.56		20.4	72.0	58.8	31.6	0.73	1.11	37.8
U-DROPm	0.65 ± 0.06	0.03	0.65	65.3	60.0	76.2	46.9	1.63	0.58	63.5
F-DROPm	0.60 ± 0.07	0.18		44.9	68.0	73.3	38.6	1.40	0.81	52.7
**Results for imaging (*n* = 350)**	**Area ± SD of ROC curve**	***p* for the area**	***p* for U vs. F**	**Sensit.**	**Specif.**	**PPV**	**NPV**	**LR+**	**LR−**	**Accur.**
U-TcpO2	0.47 ± 0.03	0.27	0.72	42.2	63.5	45.6	60.3	1.16	0.91	54.6
F-TcpO2	0.48 ± 0.03	0.55		53.1	54.2	45.6	61.5	1.16	0.87	53.7
U-DROPm	0.66 ± 0.03	<0.01	0.91	55.1	69.5	56.6	68.1	1.80	0.65	63.4
F-DROPm	0.65 ± 0.03	<0.01		55.8	69.0	56.6	68.3	1.80	0.64	63.4
**Results for imaging and symptoms (*n* = 350)**	**Area ± SD of ROC curve**	***p* for the area**	***p* for U vs. F**	**Sensit.**	**Specif.**	**PPV**	**NPV**	**LR+**	**LR−**	**Accur.**
U-TcpO2	0.46 ± 0.03	0.30	0.12	52.6	52.5	29.2	74.9	1.11	0.90	52.6
F-TcpO2	0.53 ± 0.03	0.42		49.5	51.4	27.5	73.2	1.02	0.98	50.9
U-DROPm	0.68 ± 0.03	<0.01	0.87	56.8	70.2	41.5	81.4	1.91	0.61	66.6
F-DROPm	0.69 ± 0.03	<0.01		63.2	66.7	41.4	82.9	1.89	0.55	65.7

As shown in Figure 4, the areas under ROC were not significantly different for upper arm and forearm DROPm values, but the DROPm values that provided the shortest distance to the 100% sensitivity/100% specificity angle (best compromise of sensitivity and specificity to be used for diagnosis) were not the same for U-DROPm and F-DROPm.

These threshold differences were found whatever the end point used, with thresholds obtained being −4.5 vs. −14.5 mmHg, −6.5 vs. −14.5 mmHg, −7.5 vs. −14.0 mmHg, −6.5 vs. −14.5 mmHg and −7.5 vs. −14.5 mmHg, for the detection of Symp+, US+, Ang+, Imag+ and CS + results at the upper arm vs. forearm levels respectively. Note again that the forearm DROPm value for Ang+ is provided but that the area of the ROC curve is not significant as shown in Table 2.

## Discussion

As per our hypothesis, there was a lower DROPm (larger decrease of DROP) at the forearm level than at the upper arm level as a result of the difference in altitude changes of the probes during provocative manoeuvres performed in the standing position: median values found for DROPm (see Table 2) being systematically lower by 6–10 mmHg at the forearm than at the upper arm level. Contrary to our hypothesis, diagnostic performances estimated from the AUROC were similar at the forearm and upper arm levels, but with a lower DROPm threshold value at the forearm level than at the upper arm level.

Many authors advocate that neurogenic TOS comprises over 90% of all TOS cases, while arterial TOS is rare (Sanders et al., 2007; Illig et al., 2016). Nevertheless, in many cases the classification of TOS remains difficult with intricate signs of arterial and neurogenic compression (Likes et al., 2014; Ferrante and Ferrante, 2017). Inversely, there is a high rate of positive ultrasound or angiographic results (evidence of arterial compression during provocative manoeuvres) in asymptomatic patients (Plewa and Delinger, 1998; Nord et al., 2008). This underlines the interest of improving arterial investigations in patients with suspected TOS (Likes et al., 2014; Molina and D'Cunha, 2008). Ultrasound allows for simple, low-cost and accurate recordings and remains relatively simple. It is widely used despite some debate regarding its accuracy (Sobey et al., 1993; Nord et al., 2008; Stapleton et al., 2009). Nevertheless, Doppler (as would pulse plethysmography) provides evidence for flow impairment but not for the presence of ischemia itself. Indeed, even arterial positional non-occlusive compression can provide sufficient perfusion to result in no significant oxygen delivery deficit and cause no arterial ischemia (Hersant et al., 2022). There are potential advantages of using TcpO2 to diagnose an arterial compression and argue for arterial TOS. It enables direct monitoring of oxygen availability as one of the determinants of pain and of the decline in force during ischemia (Hogan et al., 1994; Hogan et al., 1999). TcpO2 can be used during dynamic tests to provide quantitative results of regional blood flow impairment (Abraham et al., 2018a). The specific interest in the present study was the ability to objectively monitor both upper arms and forearms throughout the period of, and in the recovery period from, the provocative tests. The DROP calculation was of specific interest here to account for eventual systemic pO2 changes (as it could result from increased arterial pO2 accompanying hyperventilation due to upper limb ischemic pain). The absolute pO2 measurements that we took fall within the range of previous studies that report a mean normal TcpO2 of 52–65 mmHg at the upper limb level (Manabe et al., 2004; Babilas et al., 2008; Blake et al., 2018). From a technical point of view, it could appear surprising that analysing TcpO2 at the upper limb in patients with suspected TOS had never been performed. This probably relies on the fact that for years TcpO2 results were expressed as regional perfusion index (RPI). The transcutaneous oxygen gradient makes absolute values only fairly reliable and results in RPI being of poor reproducibility. Indeed, this gradient is unpredictable, variable from one patient to another or one probe position to another in close proximity, but constant over time for a defined probe position. The specific interest of DROP is to account only for pO2 changes over time, and as a result, to be completely insensitive to the transcutaneous oxygen gradient. Other microvascular techniques such as laser doppler or near-infrared spectroscopy are potential candidates for the future, but to the best of our knowledge have never been tested in TOS.

As expected, arm elevation induces a decrease in TcpO2 that is roughly proportional to the degree of upper limb elevation. Blake et al. (2018) observed in patients in the lying position that the mean decrease in TcpO2 during upper limb elevation of 45° above its resting level was only 1 mmHg at the upper arm level, approximately 5 mmHg at the forearm level and in excess of 15 mmHg at the hand level, while chest TcpO2 remains almost unchanged. Therefore, with the average upper limb length in adults being 70 cm, the estimated change in TcpO2 was approximately 3 mmHg for a 10 cm elevation. The amplitude of elevation from the resting position in our study was estimated at 40–45 cm for the forearm probe but only approximately 10–15 cm for the upper arm probe. Subsequently, the ∼8 mmHg difference between U-DROPm and F-DROPm observed in the prediction of Imag + results is consistent with previously cited results (Blake et al., 2018). This also suggests that any F-DROPm higher than −10 mmHg during EAST tests is expected to result only from the normal fall in TcpO2 due to elevation of the probe above heart level. This is also consistent with the optimal cut-off point that we found in predicting the presence of subclavian arterial compression on arteriography (Abraham et al., 2020). From a clinical point of view, it is unlikely that an arterial compression that would result in no ischemia could be the cause of upper limb symptoms of ischemic origin. Consequently, we believe that, since it measures the presence or absence of ischemia, TcpO2 provides unique evidence for a causal relationship between the arterial compression and the symptoms of upper limb positional pain or weakness.

We noted that the DROPm values observed in patients with positive imaging were not as low as the ones observed in our previous experiments. Nevertheless, the diagnostic performances obtained in the present study and assessed by the AUROC are very close (at both the forearm and upper arm level) to those reported in our previous studies: 0.69 ± 0.06 for arteriography (Abraham et al., 2020) and 0.69 ± 0.04 for ultrasound (Henni et al., 2019). These two observations are very likely to result from the fact that the duration of the candlestick/surrender position of a Ca-Pra manoeuvre was limited to 30 s, which possibly reduced the severity of upper limb ischemia in cases of arterial compression. Due to the relatively slow responsiveness of TcpO2 to abrupt tissue pO2 changes, it is possible that an early reperfusion occurred, preventing the TcpO2 DROP values from decreasing further. It is clear that normalising the duration of the provocative manoeuvres allows for a better inter-individual comparison, but it might have reduced the diagnostic performance of the test by limiting the DROPm value resulting from impaired perfusion. Whether, at the individual level, the provocative manoeuvre should be as long as possible to enable better discrimination between the DROPm that would result from gravity changes that would remain close to zero and abnormal DROPm results, has yet to be determined.

### There are limitations to the present work

Firstly, TcpO2 is a time-consuming technique, and beyond its interest in proving upper limb ischemia, it is uncertain that it shall be used routinely in the diagnosis of TOS. Other approaches to oxygen delivery to the upper limb could be proposed. It was previously shown that the decrease in finger saturation measured with pulse oximetry in patients with possible thoracic outlet syndrome can allow for discrimination between normal subjects and subjects with TOS, but the number of observations where the signal could not be measured (due to loss of pulsatility with complete arterial occlusion) during the dynamic manoeuvre was not reported (Braun et al., 2012). Near-infrared spectroscopy is another candidate but to the best of our knowledge, it has never been tested within the context of TOS.

Secondly, we did not compare our results to angiography in all our patients. Radiological imaging is lacking for most of our patients because it is only used as a pre-surgical approach, not as a routine investigation. Further TcpO2 measurements were not taken simultaneously to ultrasound imaging. This was intentional, so as to ensure blinded results from the two techniques. It should be pointed out that ultrasound was sometimes performed in the supine position while TcpO2 recordings were systematically carried out in the standing position. Whether or not lying down might influence the results of ultrasound investigation, as it does for angiography (Cornelis et al., 2008), has never been studied. Another concern is the fact that we retrieved ultrasound results from the report while the maneuvers used to induce a compression always included a Roos/EAST test but rarely included a Ca-Pra procedure Prayer. From the report it cannot be excluded that the investigation was considered positive because a maneuver different from the EAST/Roos test was positive, while the Roos/EAST test itself was negative. Similarly we do not know whether tests for angiography or ultrasound were performed one side at a time or on both sides simultaneously.

Thirdly, a correlation with the characteristics of clinical symptoms (pain, hand pallor, paraesthesia, *etc.*) warrants future investigations. It is possible that positive ultrasound or clinical arterial investigations do not necessarily result in ischemia, which could be an explanation for the apparent high rate of US + results reported in the literature in healthy subjects (Plewa and Delinger, 1998; Nord et al., 2008). It could also be that symptoms relate to the associated neural compression, which might explain the relatively high rate of apparent false negative TcpO2 results when compared to ultrasound. The point of specific interest here is that TcpO2 provides measurable proof of the relationship of “clinical symptoms due to documented symptomatic ischemia ... Caused by compression” (Illig et al., 2016), and therefore fills the gap between imaging (ultrasound or angiography) and symptoms used to classify patients in the arterial TOS group.

Lastly, the effects of surgical and non-surgical treatments on TcpO2 remain to be studied. It might be that conservative management allows for sufficient improvement of arterial inflow (at least as a persistent low flow) to prevent ischemia from occurring in sports practice or daily life and to improve symptoms, while classical laboratory tests in maximal abduction and inspiration remain positive for the presence of an arterial occlusion. When conservative management fails, surgery seems efficient, allowing 75%–80% of athletes to return to sports at a level at least similar to pre-surgical activity (Chandra et al., 2014; Shutze et al., 2017), and mostly within 6–10 months (Chandra et al., 2014; Thompson et al., 2017). Ultimately, the success of the operation is based on the clinical outcome after surgery in that it is the sole formal proof that the diagnostic process was valid, but only a fraction of our patients underwent surgery.

## Perspectives

From a physiological and practical point of view, the present work suggests that, when monitoring upper limb ischemia during provocative manoeuvres that include an upper limb elevation, one should account for lower DROPm values at the forearm than at the upper arm due to differences in the altitude. We believe that a measurable ischemia (estimated through DROPm calculation) may strengthen the responsibility of arterial compression in upper limb symptoms in patients with suspected TOS.

From a diagnostic point of view, the diagnosis of TOS is a holistic approach based on a combination of patients’ histories, clinical diagnostic testing and mainly ultrasound imaging. The cost-benefit of TcpO2 relative to the other diagnostic approaches used for patients with suspected TOS remains to be determined. Nevertheless, Tcpo2 might be critical in providing recordable evidence for the presence of ischemia because of arterial impaired inflow (as can be determined by radiological or ultrasound imaging). Whether portable multichannel TcpO2 devices might be of interest to confirm the presence of ischemia during usual professional or sports activities is a fascinating direction for future studies.

## Conclusion

To the best of our knowledge, this is the first report comparing upper arm and forearm TcpO2 indices in patients with suspected TOS to show upper arm or forearm DROPm show similar diagnostic performance for detecting upper limb arterial compression and/or symptoms during provocative manoeuvres. Nevertheless, for detecting positional arterial compression or symptoms, and symptoms associated with arterial compression during arm abduction, different thresholds for U-DROPm or F-DROPm must be used.

## Data Availability

The raw data supporting the conclusion of this article will be made available by the authors, without undue reservation.

## Abbreviations

Ang+/Ang−, Positive/negative angiography; Ca-Pra, Candlestick—prayer manoeuvre; CS+/CS−, Presence/Absence of positional symptoms with associated positive imaging; DROPm, Minimal decrease from rest of oxygen pressure; EAST, Elevated arm stress test; F-, Forearm; Imag+/Imag−, Positive/negative imaging (either ultrasound or angiography); ROC, Receiver operating characteristics; Symp+/Symp−, Presence/Absence of positional symptoms; TOS, Thoracic outlet syndrome; TcpO2, Transcutaneous oxygen pressure; U-, Upper arm; US+/US−, Positive/negative ultrasound imaging.
